# Successful Management of Recurrent Cholangitis Post Cholecystectomy in a Primary Care Hospital

**DOI:** 10.7759/cureus.46450

**Published:** 2023-10-04

**Authors:** Haris Khan, Muhammad Saad Irfan, Gulfan Ullah, Muhammad Afnan, Rabia Younas

**Affiliations:** 1 Medicine, Rehman Medical College, Peshawar, PAK; 2 Medicine and Surgery, Peshawar Medical College, Peshawar, PAK; 3 Botany, Kohat University of Science and Technology, Kohat, PAK; 4 Cardiovascular Medicine, Khyber Medical University Institute of Medical Sciences, Kohat, PAK; 5 General Surgery, Tehsil Headquarters (THQ) Hospital Mian Channu, Mian Channu, PAK

**Keywords:** inflammation, cholecystectomy, cholangitis, gallbladder disease, gall stones, laparoscopy

## Abstract

Cholangitis, a pathological disease characterized by inflammation of the biliary system, often occurs in conjunction with gallstone blockage and may lead to various problems, persisting for extended periods after cholecystectomy. The present report provides a comprehensive account of a clinical case involving a 35-year-old female patient who had undergone cholecystectomy three years before and is now experiencing symptoms consistent with cholangitis. The individual was originally given conservative therapy, which included the administration of intravenous ceftriaxone antibiotics, analgesics, fluids, and gastrointestinal treatment. Subsequently, they were sent to a tertiary care hospital for the performance of endoscopic retrograde cholangiopancreatography (ERCP) and the placement of a stent. Following a period of seven days, laboratory tests showed a return to normalcy, showing a positive outcome in response to the use of conservative management strategies. The patient made the decision to have an elective laparoscopic cholecystectomy, resulting in a favorable recuperation and a hospitalization period of 24 hours. The aforementioned results jointly demonstrate the efficacy of conservative therapy in treating cholangitis and the potential for eventual elective surgery in individuals experiencing prolonged gallbladder problems. In conclusion, this case underscores the need to maintain a state of alertness with respect to complications associated with cholecystectomy, such as cholangitis. It also emphasizes the effectiveness of conservative treatment approaches and the probable necessity for elective surgical intervention.

## Introduction

Cholangitis is an inflammation caused by obstruction of the biliary tree due to gallstones, some strictures, or neoplastic growth [1]. Cholangitis has a wide range of symptoms, including right upper quadrant pain, temperature, and jaundice, making up for the classic triad of symptoms, also known as Charcot’s triad. A Reynolds' pentad, wherein mental status changes and sepsis are added to the group of the triad, is likewise conceivable. Thus, broad-spectrum antibiotics and, potentially, emergency biliary tree decompression are therapeutic options for patient management [2].

A case of a patient, who undertook cholecystectomy three years ago and presented to us with the symptoms of cholangitis, is presented in this article. It highlights the importance of being aware of the potential complications of cholecystectomy, even in patients who have undergone the procedure several years prior. To the best of our knowledge, there have been no reported instances of complete cholecystectomy being performed in a primary care hospital.

## Case presentation

A 35-year-old female presented to our primary care hospital's outpatient department with a one-week history of fever, chills, and abdominal pain. The patient also experienced symptoms such as nausea, vomiting, and clay-colored stools, which provided a comprehensive picture of her condition. Upon scrutinizing her previous records, it was discovered that she underwent an open cholecystectomy three years ago. The patient had a history of systemic hypertension and diabetes mellitus. A laboratory evaluation revealed signs of systemic inflammation (Table 1).

**Table 1:** Comparison of the laboratory test results with the normal values *International units per liter.

Variables	Observed values	Normal values
White blood cells (per microliter)	14,000	4500-11,000
C-reactive protein (mg/L)	12	<10
Alkaline phosphatase (IU/L)*	150	44-147
Aspartate aminotransferase (units/L)	32	0-35
Prothrombin time (seconds)	12	12-15
Total bilirubin (mg/dl)	5.0	<1.0
Direct bilirubin (mg/dl)	4.0	<0.35
Indirect bilirubin	1.0	0.2-0.8

An abdominal ultrasound revealed the presence of a residual gallbladder with pericholecystic fluid, a positive sonographic Murphy's sign, and cholelithiasis. It is worth noting that the patient had previously undergone a cholecystectomy, yet a residual gallbladder was still evident. The common bile duct was dilated (9 mm). This was consistent with the diagnosis of acute obstructive cholangitis with cholecystitis (Video 1). The patient was put on conservative management including intravenous ceftriaxone antibiotics, analgesics, fluids, bowel care, and intake output monitoring. The patient was taken to a tertiary care hospital for endoscopic retrograde cholangiopancreatography (ERCP) and stenting. The laboratory tests performed after seven days showed typical leukocyte count and ordinary C-responsive protein with normal liver function tests. She underwent elective redo laparoscopic cholecystectomy after four weeks for residual gallbladder. The patient recovered uneventfully and was discharged after 24 hours of hospital stay.

**Video 1:** Laparoscopic cholecystectomy in a patient for residual gallbladder

## Discussion

The primary research question of the study revolves around the management of cholangitis in patients with a history of cholecystectomy. The presence of symptoms that occur after cholecystectomy is referred to as a post-cholecystectomy syndrome, which can either relate to gallbladder pathology or lead to the development of new symptoms originating from the gallbladder, potentially leading to cholangitis.

Cholangitis, an infection of the biliary tree, can arise from gallstone blockage or strictures/tumor growth, leading to a range of symptoms from mild to severe sepsis. The classic triad of symptoms, including pain, fever, and jaundice in the right upper quadrant (Charcot's triad), is indicative. Thus, broad-spectrum antibiotics and, potentially, emergency biliary tree decompression are therapeutic options for patient management [3]. Cholangitis is clinically categorized into three types: primary sclerosing (PSC), secondary (acute), and IgG4-associated cholangitis (IAC), with both hereditary and acquired factors contributing to its development [4].

Our case report summarizes the patient with acute cholangitis whose major causes include biliary tract blockage, high intraluminal pressure, and bile infection. A biliary system colonized by bacteria yet unblocked does not usually lead to cholangitis. Actually, biliary blockage is thought to reduce human antibacterial defenses, promote immunological dysfunction, and increase small bowel bacterial colonization. Although the specific method is unknown, the retrograde ascent is the way in which germs are thought to enter the biliary system through the duodenum or portal venous blood [5].

As a result, the infection spreads to the hepatic ducts and causes severe illness. Bacteremia (25%-40%) results from increased biliary pressure, which leads to infection into the biliary canaliculi, hepatic veins, and perihepatic lymphatics. Choledocholithiasis is the most prevalent cause of acute cholangitis, which is followed by ERCP and tumors.

All those disorders such as benign or malignant constraints, extrinsic pancreatic blockage, or parasitic infections that cause bile blockage or stasis in the common bile duct (CBD) may result in bacterial infection and cholangitis. Partial blockage is linked with a greater risk of infection than total blockage [6].

Several factors such as early recognition, the response of a patient to therapy with other medical conditions, and treatment of cholangitis lead to the prognosis. Underlying diseases are one of the predisposing factors to the high mortality of patients with cholangitis. The advent of therapeutic endoscopic sphincterotomy, stone extraction, endoscopic retrograde cholangiography, and biliary stenting has decreased mortality from 100% to approximately 5%-10%.

## Conclusions

The main research question is how to manage cholangitis in a patient with a history of cholecystectomy. The research suggests that employing ERCP followed by stenting is an effective approach for addressing biliary obstruction in these cases. The study underscores the significance of advanced medical interventions and multidisciplinary approaches to achieve favorable outcomes. By presenting a case study, this research contributes valuable insights into the existing literature on post-cholecystectomy cholangitis, shedding light on the unique challenges and successful management strategies to achieve favorable outcomes.

## References

[1] Rajput D, Gupta A, Gupta S, Kumar S (2021). A series of biliary tract cancer with coexistent non-biliary second malignancy from sub-himalayan region of India. Cureus.

[2] Mohammad Alizadeh AH (2017). Cholangitis: diagnosis, treatment and prognosis. J Clin Transl Hepatol.

[3] Chen TS, Lin XH, Peng YL, Luo JC, Chen YT, Hou MC, Lee FY (2017). Cholecystectomy decreased the recurrent cholangitis after clearance of bile duct stones by ERCP in patients with gallstone-related cholangitis. J Chin Med Assoc.

[4] Rosing DK, De Virgilio C, Nguyen AT, El Masry M, Kaji AH, Stabile BE (2007). Cholangitis: analysis of admission prognostic indicators and outcomes. Am Surg.

[5] Park CH (2018). [The management of common bile duct stones]. Korean J Gastroenterol.

[6] Kumar M, Sonika U, Sachdeva S, Dalal A (2021). Mucin-filled CBD, difficult to manage cholangitis. BMJ Case Rep.

